# Pediatric Overweight, Fatness and Risk for Dyslipidemia Are Related to Diet: A Cross-Sectional Study in 9-year-old Children

**DOI:** 10.3390/nu15020329

**Published:** 2023-01-09

**Authors:** Paulo Mascarenhas, José M. Furtado, Sílvia M. Almeida, Maria E. Ferraz, Fernando P. Ferraz, Pedro Oliveira

**Affiliations:** 1 Centro de Genética Médica e Nutrição Pediátrica Egas Moniz, Instituto Universitário Egas Moniz, 2829-511 Monte de Caparica, Portugal; 2 Department of Population Studies, ICBAS—Instituto de Ciências Biomédicas Abel Salazar, University of Porto, 4050-313 Porto, Portugal

**Keywords:** children, western diet, overweight, obesity, dyslipidemia

## Abstract

Pediatric overweight, dyslipidemia and insulin resistance can result from unhealthy lifestyle habits and increase morbidity and mortality in adulthood. Herein, we evaluated the relationship between diet and physical activity patterns with the metabolic health of 9-year-old school children. Measurements included anthropometry, adiposity, lipid, and glycemic profiles. Questionnaires evaluated diet and physical activity. Exploratory factor analysis (EFA) screened for diet patterns, and multilevel models evaluated diet and physical activity patterns against overweight, dyslipidemia, and insulin resistance markers across schools and children. EFA highlighted two diet patterns, Western and Traditional. Food rich in fat, salt, and sugar and fewer vegetables and fruits defined the Western pattern. The Traditional pattern, linked to healthier eating habits, had analogies to the Mediterranean diet. Overall, 39% of the children were overweight (including the obese), while 62% presented cardiovascular risk factors on their lipid profiles. Normal-weight children presented 60% high cholesterol incidence. Global insulin resistance incidence was 4.1%, but almost doubled among the overweight/obese. The Westernized diet consistently linked to worse cardiovascular risk markers, even independently of physical practice. Intensive or competitive physical activity was associated with decreased triglycerides (*p* = 0.003), regardless of diet. Future prospective studies are warranted to validate these results externally.

## 1. Introduction

Pediatric overweight is a major factor in the adulthood obesity epidemic, translating into an increased mortality and morbidity burden in adult life. Characterised by excessive fat accumulation, overweight is influenced by genetic and environmental factors, including diet and physical activity (PA) [1].

While the health consequences of pediatric overweight are still poorly studied compared to adults, pediatric overweight has been consistently reported as a key risk factor for future metabolic conditions, such as type 2 diabetes mellitus, metabolic syndrome, cardiovascular diseases, and certain types of cancer [2,3,4,5,6,7,8,9]. Therefore, pediatric overweight can be deleterious to quality of life [10], with associated emotional and behavioural problems due to children acquiring the cultural values of beauty and aesthetics considerably before puberty. This acknowledgement leads obese children to low self-esteem, sadness, loneliness, and nervousness [11,12,13].

The development of overweight in children and adolescents is complex, with several risk factors and interrelated mechanisms. Among the environmental components of overweight and obesity, lifestyle habits, such as little or no PA, unbalanced eating habits, and sleeping disorders, seem to be decisive determinants [9,14,15,16]. Some socio-economic and cultural factors also impact children’s health. Screen time, inconsistent sports practice, and unhealthy food habits seem to be positively associated with lower socio-economic status, as assessed by parental education and wealth indicators [17,18,19]. Furthermore, ethnic disparities regarding overweight [20] and the parental misperception of childhood excess weight [21] may also play a role. Lately, the COVID-19 pandemic imposed worldwide measures, such as lockdowns, leading to even longer children’s sleep time, leisure-based screen activity, unhealthy food choices, and sedentarism [22,23,24].

Two major metabolic complications of childhood increased adiposity, decisive to the future cardiovascular condition, are the early onset of insulin resistance (IR) and the dyslipidemia phenotype [25,26]. Therefore, the contribution of dietary habits and PA to overweight and metabolic disorders in children is of major importance because these are key modifiable behaviours [27,28].

Regarding nutritional intake and dietary habits, European children seem to maintain a hypercaloric fast-food diet and poorly balanced meals, and Portuguese children are no exception [29,30]. This trend seems to worsen as the child approaches adolescence and becomes more autonomous, accessing junk food in the vicinity of schools easier [31,32,33].

Similarly, PA seems also to be neglected, despite being an important preventative factor for the risk of being overweight in children and adolescents [34]. At the same time, sedentary behaviours, often concomitant with the intake of hypercaloric foods, also prevail in children and adolescents [35]. Young people spend approximately 60% of their awake time sitting, thus making this the most common sedentary habit [36,37]. Remarkably, studies report an increase in sedentary behaviour around early adolescence (11 to 13 years old), mostly in front of a screen [15,38,39]. In girls, a phenomenon called ‘exercise embarrassment’ makes physical activity decline during adolescence, most profoundly among minorities [40]. Proposed strategies to fight sedentary behaviour and, by proxy, overweight in children include the removal of screens from children’s bedrooms and intensifying physical practice [36].

Diet, PA, and sedentary behaviour cluster together in complex ways that are not always well understood, leading to controversy regarding the magnitude of the individual contributions of these lifestyle risk factors to overweight and metabolic disorders in children [28].

Herein, we aim to evaluate the relationship between lifestyle habits and the metabolic health of 9-year-old children, sample screening for features of dyslipidemia and IR. In addition, we show that diet and physical practice are linked early in life with overweight, adiposity, and serum metabolic markers.

## 2. Materials and Methods

### 2.1. Study Design and Participants

This study was prepared under the Strengthening the Reporting of Observational studies (STROBE) guidelines [41] and STROBE-nut extension [42]. Data originated from a cross-sectional study of prepubertal children, carried out between 2012 and June 2013, in 20 public schools from urban areas of the Lisbon and the Tagus Valley metropolitan region (central Portugal).

### 2.2. Anthropometric and Bioelectrical Impedance Analysis

Two pediatric consultants performed all anthropometrical measurements in each school gym, using a previously described protocol [43], provided in the supplementary file. Weight, height, body mass index (BMI), BMI *z*-score (zBMI), waist circumference (WC), hip circumference (HC), mid-upper arm circumference (MUAC), calf circumference (CC), and percentage of body fat (%BF) were obtained from participants dressed in lightweight clothing and without shoes. The waist–hip ratio (WHR [WC/HC]) and waist-circumference–to–height ratio (WHtR [WC/height]) anthropometric indices were calculated. Children were categorised as normal weight or overweight/obese, according to the World Obesity/Policy and Prevention standards [formerly International Obesity Task Force (IOTF)] cut-offs [44].

### 2.3. Biochemical Analysis

Participants were instructed to fast overnight before venipuncture for early morning blood sampling. The following serum biochemical parameters were measured: total cholesterol (TC), high-density lipoprotein (HDL-c), low-density lipoprotein (LDL-c), oxidised LDL-c (oxLDL-c), triglycerides (TG), apolipoproteins A1 (ApoA1) and B (ApoB), glucose, and serum insulin. All assessments were made using previously described methods [45] (provided in the supplementary file). The following formula estimated the Homeostasis Model Assessment of Insulin Resistance (HOMA-IR): [insulin (µU/mL) × glucose (mg/dL)/405]. To categorise children regarding the evaluated serum lipidic and apolipoprotein profile concentrations and calculate the associated percentages, we adopted the acceptable ranges proposed by De Jesus et al. [46]. Values for plasma lipid and lipoprotein levels were from the National Cholesterol Education Program (NCEP) Expert Panel on Cholesterol Levels in Children and the apolipoprotein ranges from the Third National Health and Nutrition Examination Survey (NHANES III) [47,48]: TC < 170 mg/dL; HDL-c > 45 mg/dL; LDL-c < 110 mg/dL; TG < 75 mg/dL; ApoB < 0.9 g/L and ApoA1 > 1.2 g/L. For HOMA−IR in prepubertal children, we adopted the superior limit cut-offs before insulin resistance proposed by Kurtoglu et al. 2010 [49]: 2.67 for boys and 2.22 for girls. We labelled every biochemical result as abnormal or unhealthy when outside the limits defined by the adopted acceptable boundaries.

### 2.4. Diet and Physical Activity Surveys

Children were asked to answer two previously validated Likert-scaled self-report questionnaires [50,51], one regarding food frequency (FFQ) and the other about physical activity frequency (PAFQ). This task was performed in the classrooms under the teacher’s supervision.

The FFQ assessed the weekly intake frequency of thirty-three types of food and beverage items commonly consumed in Portugal (Table S1). Participants were asked to select between four increasing levels of consumption frequency: never, 1–3 per week, 4–6 per week, and every day of the week. Due to the non-quantitative nature of the form, no food intake measures beyond weekly consumption frequency were assessed.

The second questionnaire screened the children’s PA frequency in the past week, before and after school, and over the weekend. It comprised two distinct parts. In the first, questions evaluated the frequency of activities, such as general practice (Q1), rest and leisure time (Q2), prolonged PA (Q3), high-intensity PA (Q4), and competitive sports activity (Q5). The following section evaluated the weekly frequency of 17 sports (such as soccer) and everyday soft PAs (such as walking). An additional field, designated as ‘others’, accounted globally for the weekly frequency practice of every missing sport/activity in the survey list. Therefore, study participants were categorised in increasing levels from ‘never’ to ‘every day’ of the last week. Thus, the second part of the PAFQ allowed us to estimate the overall frequency and diversity of practised sports.

### 2.5. Statistical Analysis

The descriptive statistic and the exploratory factor analysis (EFA) were performed with SPSS for Windows (SPSS Inc., Chicago, IL, USA, version 26). Statistics for anthropometric and clinical analysis panels were calculated by sex and IOTF categories, with standard deviations (SD) displayed next to mean results. Differences between means were t-tested with Dunn–Bonferroni adjustment after statistical assumptions confirmation.

Regarding the FFQ results, to better screen for dietary patterns (DPs), the food and beverage items were grouped into seventeen (17) food groups (as detailed in Table S1), based on similarities of biochemical constituents and nutrient profiles. The food groups’ overall frequency per week was the cumulative consumption frequency of the included food and beverage items. The food groups’ internal consistency was evaluated through Cronbach’s alpha (α). Next, we performed EFA to screen for food group clusters (factors) that accounted for the largest variance among the surveyed children’s overall diet and in which consumption frequencies were correlated. To evaluate the EFA sampling, we performed the Kaiser–Meyer–Olkin (KMO) test, and for the correlation matrix suitableness, we performed Bartlett’s test of sphericity. We considered KMO > 0.8 adequate sampling and a significant result in Bartlett’s test (*p* < 0.05) as a good EFA prognosis for our dataset. To make data interpretation easier and fulfil posterior analysis assumptions, we standardised factors and minimised their intercorrelation by applying Varimax rotation with Kaiser normalisation.

The selection of two clusters of food groups after EFA was determined by the Kaiser criterion (eigenvalues above 1), the scree plot line inflexion, and the acknowledgement of a recognisable DP. Factor loadings were used to measure the strength and direction of the association between the food groups and the two selected DPs. Next, standardised scores (ranging from −1 to 1) were calculated for each child, using a multiple regression approach and grouped in tertiles for each DP. Child diets scoring close to 1 (3rd tertile) meant a strong association with the DP, while scoring close to −1 (1st tertile) had the opposite meaning. In order to discriminate better if each child’s food consumption frequency, as evaluated by the scores, was more related to one or the other DP, the two DP scores were subtracted and rescaled to obtain an additional combined score. Anthropometric, adiposity, and biochemical variables statistics were then calculated against the scores tertiles, and the mean differences *t*-tested with Dunn–Bonferroni adjustment.

Due to the hierarchical structure of our data, we evaluated the relationship of the children’s sex, diet, and PA as independent variables, with zBMI, %BF, and circulating markers as outcomes, through a two-level (school and individual child) multilevel regression analysis. For each model, the school level included as a nested variable the average purchasing power per capita (APPPC) of the school, involving region (municipality) at the measurement’s year to discriminate for eventual socio-economic differences. The APPPC was obtained from PORDATA [52], an open-access platform, from which we can collect annual socio-economic indicators for each Portugal region. The regression models were fitted through the lmer function included in the lme4 package for R framework (version 4.1.0) [53]. The model’s results included the variables’ coefficients, estimated through restricted maximum likelihood (REML), and the children and the school district region level’s random effects variances. Variables or levels with significant positive coefficients were interpreted as being associated with increased outcome values and the opposite if significant negative coefficients were present.

A two-sided *p* < 0.05 defined the significance of all performed statistical analyses.

## 3. Results

A total of 499 children (245 girls and 254 boys) had anthropometric and bioelectrical impedance measurements. Regarding ethnicity, most children were Caucasian (93%, n = 466). After informed consent was provided, 387 had blood collected for further analysis, and out of these, 272 answered the nutritional and physical activity surveys (lifestyle assessment). Both whole and lifestyle assessment samples had no differences for the possibility of selection bias (*p* < 0.05). The mean age of study participants was 9.4 (±0.5) years (Table 1).

### 3.1. Anthropometry and Bioelectrical Impedance

According to IOTF, 39% of the children were overweight/obese, with significant differences between boys and girls regarding WHR (*p* < 0.001) and impedance measurements (*p* = 0.031). Girls showed higher %BF (*p* = 0.031), but lower WHM (*p* < 0.001); yet, boys had significantly higher RMR (*p* < 0.001). Overweight children presented significantly higher anthropometric measures (*p* < 0.05) and %BF (*p* < 0.001), but not in skeletal muscle (%) (*p* = 0.613). Furthermore, both male and overweight children had significantly increased RMR values (*p* < 0.001). Overweight/obese children showed significantly decreased weight-adjusted RMR (31.1 Kcal/Kg·day) than normal-weight (38.8 Kcal/Kg·day) (*p* < 0.001). Weight-adjusted RMR was slightly reduced in girls compared to boys (34.5 Kcal/Kg·day versus 35.7 Kcal/Kg·day, respectively).

### 3.2. Biochemical Analysis

Regarding the lipidic profile results (Table 2), girls showed significantly higher results for LDL-c (*p* = 0.045), ApoB (*p* = 0.010), TC/HDL (*p* = 0.024), LDL/HDL (*p* = 0.010), ApoB/ApoA1 (*p* = 0.009), and oxLDL-c (*p* = 0.002), and lower results for HDL-c (*p* = 0.023) and ApoA1 (*p* = 0.011). Regardless of sex, overweight/obese children presented increased lipidic serum profiles (except for HDL-c and ApoA1) than normal-weight counterparts. This consistent increase was significant for LDL-c (*p* < 0.001), TG (*p* < 0.001), ApoB (*p* < 0.001), TC/HDL (*p* < 0.001), LDL/HDL (*p* < 0.001), ApoB/ApoA1 (*p* < 0.001), and oxLDL-c (*p* = 0.022). Finally, HDL-c and ApoA1 were significantly lower among the overweight/obese (*p* < 0.001 and *p* = 0.002, respectively).

Considering the TC pediatric reference threshold (TC < 170 mg/dL), about 1:2 children observed were at risk for dyslipidemia, regardless of zBMI grade. Regarding the other lipidic profile markers, the abnormal results were 12.2% (HDL-c), 31.8% (LDL-c), 11.1% (TG), 14.6% (ApoB), and 26.3% (ApoA1). Boys and girls did not differ in the glycemic profile. However, there were significantly higher levels of glucose (*p* = 0.010), insulin (*p* < 0.001), and HOMA-IR (*p* = 0.008) in the overweight/obese children, with an average insulin resistance increase of 89%.

### 3.3. Diet Patterns Analysis

The internal consistency of EFA using the dietary frequency of 17 food groups of 272 children was acceptable (Cronbach’s α = 0.625). The KMO test result (0.7) was slightly below the adequate sampling boundary; however, Bartlett’s test indicated a good dataset prognosis. Factor loadings are displayed only when above 0.3 or below −0.3. This approach allows simplicity and points out the food groups mainly associated with each DP.

The analysis revealed two main dietary patterns (Table 3). On the one hand, the Western diet pattern (WDP) contained diet features associated with westernised ‘fat, salt and sugar’ unhealthy food habits and being poorly associated with important healthy food groups, such as vegetables and fruit. It included moderate to high loadings for food groups such as chips and salt sticks, fast food, meat (processed), sweets, bread, and fats. On the other hand, the Traditional diet pattern (TDP) included some features of the typical Mediterranean diet, and it was positively correlated with soup, fruit (or fresh fruit juice), vegetables, and water, and poorly related to some obesity-associated food groups (fried potatoes, cake or cookies, and sugar-sweetened beverages). Nonetheless, Western and Traditional both share a high frequency of red meat, poultry, and fish intake, although fish was slightly more associated with the TDP and red meat and poultry with the WDP. Three food groups had high negative loadings in the Traditional pattern, but not for the Western pattern (Table 3, WDP), and therefore were negatively related to the TDP: fried potatoes, cake and cookies, and sugar-sweetened beverages. Cereals, as well as milk and dairy products, scored low on factor loadings for both DPs. Both diet patterns explained 29.2% of the total variance, mostly attributed to WDP (18.9%).

We then explored the cardiovascular risk factors distribution by dietary pattern tertiles (WDP, TDP, and combined TDP_WDP) (Table 4). Regarding TDP_WDP, a child scoring close to 1 (3rd tertile) meant a diet associated with WDP and poorly related to TDP. A score close to −1 (1st tertile) meant the opposite. Scoring high for the WDP was associated with higher values of zBMI (*p* = 0.002), LDL/HDL (*p* < 0.001), and LDL/ApoB (*p* < 0.001) and decreased levels of ApoA1 (*p* < 0.001). On the contrary, scoring high for the TDP was related to low %BF levels (*p* < 0.001), although without significant changes in the lipid profile. No meaningful effect on the glycemic profile was found for Western or Traditional DPs. Curiously, scoring high for the combined TDP_WDP meant significantly elevated %BF (*p* < 0.001) and LDL/ApoB (*p* < 0.001), and lower levels for ApoA1 (*p* < 0.001).

### 3.4. Multilevel Analysis

Except for TG, the school level contributed little to the total variance explanation (Table 5). Nonetheless, school distribution explained 37% of TG variance. In contrast, zBMI, %BF, and the remaining variables seemed homogeneous between the studied schools. The school-level socio-economic influence for all outcomes was unexpressive, estimated through the APPPC indicator.

Boys showed a significant decreasing effect on %BF (*p* = 0.046) and LDL/HDL (*p* = 0.008) at the children’s level. The WDP was associated with increased LDL/HDL (*p* = 0.030) and LDL/ApoB (*p* = 0.008) levels. This relationship stayed significant for LDL/ApoB (*p* = 0.025) when adjusted for %BF and zBMI. On the contrary, TDP_WDP was associated with higher zBMI (*p* = 0.025) and %BF (*p* = 0.013) values. According to Q1 (general practice), about 45% of the surveyed children reported the daily practice of some degree of physical activity. The extra-curricular physical practice was significantly associated with lower TG regarding intensive and competition sports frequency (*p* = 0.003), even after adjusting for %BF and zBMI (*p* ≤ 0.006).

## 4. Discussion

The present study examines in children the relationship between diet and physical practice and risk factors for cardiovascular diseases.

At the time of this study’s data collection (2012–2013), Portugal presented a decreasing trend of the pediatric overweight prevalence, estimated at −1.2% per year, but still one of the highest rates of obesity among European countries [17,54,55]. This trend, monitored between 2008 and 2019 by the Portuguese branch of the European Childhood Obesity Surveillance Initiative (COSI Portugal), was also reported by other developed countries [9].

In Portugal, this change was probably the consequence of three main factors. First, the implementation of sugar tax legislation led to an 11% reduction in sweetened beverages’ energy intake consumption [56]. Second, the enforcement by the national authorities of a national programme promoting policies favouring PA in urban spaces [57]. Lastly, with mixed effects on childhood obesity, the 2008 global economic recession [58].

Our results showed considerable excess weight–to–height prevalence (39%) and concerning levels of adiposity (29% body fat for overweight/obese, on average) in our children sample. In its 2013 scientific report, COSI Portugal revealed a similar pediatric overweight rate (38%) for 6- to 8-year-old children in the same region sampled in our study [59].

We found that the overweight/obese displayed consistently worse mean levels for all lipids and lipoproteins we screened. Among the primary cardiovascular markers, we found elevated levels of TC in 47% of our children. On average, the major fraction of TC was LDL-c (56%). Furthermore, we found elevated levels of LDL-c in 32% of the children and significantly increased values in the overweight/obese cluster. Still, only 40% of the children showing high cholesterol were overweight or obese, leading to 60% being ‘hidden’ behind normal BMI at the measurement time. Regarding the whole lipidic profile, 62% of the children presented at least one cardiovascular risk factor (mostly high cholesterol). Studies targeting pediatric populations in Portugal, Central Europe, and Colombia, equally looking for cardiovascular risk factors in overweight/obese children, reported abnormal lipid profiles results (mostly TC and TG) in 42–50% of the children and worse average results associated with overweight or obesity [60,61,62,63].

In regards to sex, girls showed higher levels of LDL-c, ApoB, TC/HDL, LDL/HDL, ApoB/apoA1, and oxLDL-c, and lower HDL-c and ApoA1. This contrast is probably a consequence of the natural fluctuations in lipids and lipoproteins concentrations occurring with growth and maturation, which impact girls earlier. It highlights the importance of developing lipid and lipoprotein threshold concentrations for children adjusted for age and sex.

The chronic IR condition is recognised as a cardinal trigger of type 2 diabetes mellitus and cardiovascular diseases [64]. While traditionally considered mostly an adulthood disease, the type 2 diabetes mellitus demography has been changing towards increasing prevalence among young people [65,66]. We used HOMA-IR, a validated surrogate measure of IR in obese children and adolescents [67,68], to look for signs of at-risk children in our study population. We found results above the adopted thresholds in 4.1% of our children (2.7% in boys and 5.6% in girls), with the overweight/obese displaying measurements increased on average by 89%. Two conditions may cumulatively explain most of our numbers at this age: prepuberal IR, followed by puberty onset. The typical start of prepuberal IR is ∼3–4 years before the pubertal onset, and it is an eventual sign of early evolving diabetes resulting from the cumulative actions of fatness, rising insulin-like growth factor 1 (IGF-1), and adrenal hormones [69]. The pubertal endocrine maturation reportedly begins around 8–9 years in girls and 1–2 years later in boys, but being overweight may trigger IR earlier because fat is known to reduce insulin action. Then, puberty’s anatomical and physiological changes can lead to transient IR peaks [69,70,71]. Peplies et al. [72], in a longitudinal study on European children (IDEFICS cohort), found an overall prevalence of 17.8% of IR, and that overweight and obesity were the main determinants of insulin resistance. The IR rate discrepancy towards our results was probably due to the different IR adopted cut-offs.

On the dietary survey results, we could identify two DPs commonly observed in Portugal (WDP and TDP), each presenting distinct relationships to most food groups. In line with other studies [73,74,75,76,77,78,79,80,81], a healthy versus an unhealthy DP arose from the EFA, that is, TDP versus WDP. For instance, Davison et al. [75] found the ‘fruit and vegetables’ concomitantly with the ‘snacks’ eating pattern in 9- to 11-year-old children from New Zealand, while Kourlaba et al. [77] found, among others, two patterns designated in their report as ‘vegetarian/healthy’ and ‘junk food’ rich in fast foods.

Our results show zBMI, %BF, and some lipid profile markers (including HDL, ApoA1, ApoB/ApoA1, TC/HDL, LDL/HDL, and oxLDL-c) consistently getting worse across the WDP or TDP_WDP tertiles’ increasing sequence. When comparing the first and last tertiles, there were significantly higher levels for zBMI, %BF, LDL/HDL, and LDL/ApoB, and decreased ones for ApoA1. On the one hand, excess weight and abnormal lipid indicators prevailed in children following the WDP. On the other hand, children whose diets were closer to the TDP were, on average, less fat and presented healthier lipid profiles.

We also analysed the combined effect of DPs and extra-curricular PA, as children’s lifestyle features adjusted according to sex and socio-economic level of the school’s surroundings, on outcome variables representing corpulence (zBMI), fatness (%BF), lipidemia (TG, TC, LDL/HDL, LDL/ApoB), and IR (HOMA-IR). The WDP was associated with higher zBMI and %BF values and increased cardiovascular disease risk markers (LDL/HDL and LDL/ApoB), independently of %BF and zBMI. The TDP seemed to protect against fatness. These results were similar to many others studying the association of DPs with cardiovascular risk factors in children and adolescents from different countries [82], and in animal models [83]. Most of these studies reported significant associations between the Western diet and cardiovascular risk factors, such as obesity, increased IR, TG, or TC, and between Traditional analogue patterns (including the Mediterranean diet) and a healthier cardiovascular profile [50,80,81,84,85,86]. Therefore, diet changes toward reducing unhealthy food intake in children and adolescents can result in improved plasma lipid profiles that, if carried into adult life, can potentially reduce atherosclerotic vascular disease [87].

Regarding the extra-curricular physical practice, our results showed that it was significantly associated with decreased TG if intensive or competitive, independently of diet, %BF, and zBMI. This association probably resulted from the known physical exercise TG-reducing effect, previously reported in overweight/obese children and adolescents submitted to physical practice intervention programs [88], or in all children if intervention programs lasted longer than 6 months [89].

It is noteworthy to discuss some limitations presented in this study. First, not all environmental and lifestyle confounders were accounted for (e.g., fast food in the vicinity of schools and families’ wealth), as well as individual genetic susceptibility, resulting in a certain unexplained variation. Second, with an observational cross-sectional design, this study only provides clues on the relationships between overweight/metabolic problems and relevant factors; thus, further validation is required to understand these relationships better and assess temporality or causality. Third, self-reports from 9-year-old children in surveys are always susceptible to a certain level of bias, even under the teacher’s supervision. Fourth, blood drawing from children at school facilities is sometimes challenging and may have limited the sample size. Fifth, the APPPC status by region was insensitive to socio-economic differences between schools from the same region and between children within schools. Finally, due to the variability of pediatric references and cut-offs to categorise children’s weight and metabolic status according to age and sex, we expect heterogeneous comparisons with other studies.

## 5. Conclusions

This study reports a high prevalence of overweight/obesity and abnormal lipid profile in this pediatric population. Among children categorised as of normal-weight, 60% showed high hypercholesterolemia. Two diets were identified, a Westernized (linked to unhealthy habits) and a Traditional (linked to healthier eating habits). The Westernized diet was consistently linked to worse cardiovascular risk markers. Future prospective studies are warranted to validate these results externally.

## Figures and Tables

**Table 1:** Descriptive characteristics of the study population by sex and IOTF category.

	Sex				IOTF zBMI Grades					
	Female		Male		Normal		Overweight/Obese		Overall	
	*N*	Mean (SD)	*N*	Mean (SD)	*N*	Mean (SD)	*N*	Mean (SD)	*N*	Mean (SD)
Age (years)	245	9.4 (0.5)	254	9.4 (0.6)	303	9.4 (0.5)	196	9.4 (0.5)	499	9.4 (0.5)
**Anthropometry**										
Weight (Kg)	245	33.9 (7.7)	254	33.9 (8.0)	303	**29.2 (4.0)**	196	**41.1 (6.8)**	499	33.9 (7.8)
Height (m)	245	135.8 (7.0)	254	136.6 (6.9)	303	**134.4 (6.7)**	196	**139.0 (6.5)**	499	136.2 (7.0)
BMI (Kg/m^2^)	245	18.2 (3.0)	254	18.0 (3.1)	303	**16.1 (1.3)**	196	**21.2 (2.4)**	499	18.1 (3.1)
zBMI	245	0.63 (1.17)	254	0.66 (1.27)	303	**−0.15 (.75)**	196	**1.88 (.64)**	499	0.65 (1.22)
WC (cm)	236	62.9 (7.9)	253	63.1 (8.2)	296	**58.3 (3.8)**	193	**70.3 (7.4)**	489	63.0 (8.1)
HC (cm)	236	71.5 (7.6)	253	70.5 (7.8)	296	**66.4 (4.6)**	193	**78.0 (6.1)**	489	71.0 (7.7)
WHR (WC/HC)	236	**0.88 (0.05)**	253	**0.90 (0.06)**	296	**0.88 (0.05)**	193	**0.90 (0.06)**	489	0.89 (0.05)
WHtR (WC/height)	236	0.46 (0.05)	253	0.46 (0.05)	296	**0.43 (0.03)**	193	**0.51 (0.05)**	489	0.46 (0.05)
MUAC (cm)	236	20.8 (2.7)	253	21.2 (3.1)	296	**19.5 (3.2)**	193	**23.3 (2.1)**	489	21.0 (2.5)
CC (cm)	236	28.1 (3.0)	253	28.5 (4.1)	296	**26.6 (3.1)**	193	**30.9 (2.6)**	489	28.3 (3.6)
**Bioelectrical impedance**										
%BF	233	**22.78 (7.7)**	250	**21.3 (7.3)**	290	**17.1 (4.5)**	193	**29.2 (4.9)**	483	22.0 (7.5)
%SM	233	**30.7 (2.2)**	250	**31.7 (2.9)**	290	31.3 (2.9)	193	31.1 (2.2)	483	31.2 (2.6)
RMR (Kcal/day)	233	**1168 (109)**	250	**1212 (120)**	290	**1132 (86)**	193	**1280 (99)**	483	1191 (117)

Bold values highlight the statistically significant differences between Female versus Male and Normal versus Overweight/Obese (*p* < 0.05). Results include sample size (N), mean and standard deviation (SD). BF body fat, BMI body mass index, CC calf circumference, HC hip circumference, IOTF International Obesity Task Force, MUAC mid-upper arm circumference, RMR resting metabolic rate, SM skeletal muscle, WC waist circumference, WHR waist-hip ratio, WHtR waist circumference-to-height ratio, zBMI BMI *z*-score.

**Table 2:** Descriptive biochemical characteristics by sex and IOTF category.

	Sex				IOTF zBMI Grades					
	Female		Male		Normal		Overweight/Obese		Overall	
	*N*	Mean (SD)	*N*	Mean (SD)	*N*	Mean (SD)	*N*	Mean (SD)	*N*	Mean (SD)
**Lipid panel**										
TC (mg/dL)	188	172.0 (28.8)	203	170.9 (27.4)	234	170.6 (29.3)	153	172.2 (25.6)	387	171.3 (27.9)
LDL-c (mg/dL)	188	**99.2 (23.8)**	203	**94.3 (24.0)**	234	**93.2 (23.4)**	153	**101.8 (24.0)**	387	96.6 (24.0)
HDL-c (mg/dL)	188	**58 (11)**	203	**60 (12)**	234	**61 (12)**	153	**56 (11)**	387	59 (12)
TG (mg/dL)	188	66 (26)	203	61 (26)	234	**58 (22)**	153	**71 (31)**	387	63 (27)
ApoA1 (g/L)	188	**1.34 (0.19)**	203	**1.39 (0.22)**	234	**1.39 (0.22)**	153	**1.32 (0.19)**	387	1.36 (0.20)
ApoB (g/L)	188	**0.76 (0.15)**	203	**0.72 (0.14)**	234	**0.72 (0.15)**	153	**0.77 (0.15)**	387	0.74 (0.15)
TC/HDL	188	**3.06 (0.62)**	203	**2.92 (0.60**)	234	**2.85 (0.51)**	153	**3.19 (0.70)**	387	2.99 (0.62)
LDL/HDL	188	**1.79 (0.57)**	203	**1.64 (0.57)**	234	**1.58 (0.48)**	153	**1.92 (0.64)**	387	1.71 (0.58)
ApoB/ApoA1	188	**0.58 (0.15)**	203	**0.53 (0.16)**	234	**0.52 (0.16)**	153	**0.59 (0.17)**	387	0.55 (0.17)
LDL/ApoB	188	130.08 (15.66)	203	129.72 (15.46)	234	129.63 (11.68)	153	130.19 (19.63)	387	129.8 (15.58)
oxLDL-c (mU/L)	83	**7.20 (1.50)**	74	**6.39 (1.65)**	71	**6.33 (1.41)**	86	**7.22 (1.77)**	157	6.73 (1.63)
**Glycemic panel**										
Glucose (mg/dL)	188	80.4 (10.4)	203	81.9 (9.6)	234	**80.2 (9.5)**	153	**82.8 (10.6)**	387	81.2 (10.0)
Insulin (µU/mL)	83	4.84 (3.30)	74	4.08 (4.39)	71	**3.13 (1.83)**	86	**5.60 (4.56)**	157	4.48 (3.86)
HOMA-IR	83	0.94 (0.73)	74	0.78 (0.94)	71	**0.58 (0.39)**	86	**1.09 (1.02)**	157	0.86 (0.84)

Bold values highlight the statistically significant differences between Female versus Male and Normal versus Overweight/Obese (*p* < 0.05). Results include sample size (N), mean and standard deviation (SD). ApoA1 apolipoprotein A1, ApoB apolipoprotein B, HDL-c high-density lipoprotein cholesterol, LDL-c low-density lipoprotein cholesterol, oxLDL-c LDL-c oxidised fraction, TC total cholesterol, TG triglycerides, HOMA-IR Homeostatic Model Assessment for Insulin Resistance, IOTF International Obesity Task Force, zBMI BMI *z*-score.

**Table 3:** EFA results on the dietary frequency.

Food Groups	Diet Patterns	
	WDP	TDP
Chips and salt sticks	0.728	
Compound fast food	0.671	
Processed meat	0.654	
Sweets	0.645	
Bread	0.518	
Oils and fats	0.477	
Red meat and poultry	0.628	0.356
Fish	0.389	0.504
Soup		0.538
Fruits and fresh fruits juice		0.417
Water		0.479
Vegetables		0.473
Fried potatoes		−0.459
Cake and cookies		−0.399
Sugar-sweetened beverages		−0.396
Cereals		
Milk and dairy products		

TDP Traditional diet pattern, WDP Westernized diet pattern. Varimax rotation with Kaiser normalisation converged in 5 iterations. Total variance explained 29.2% (first two factors). KMO = 0.7, Bartlett’s test *p* = 0.02, and Cronbach’s α = 0.625. Factor loadings < 0.3 were not listed for simplicity.

**Table 4:** Mean (SD) descriptive results by diet pattern scores ascending tertiles.

		WDP			TDP			TDP_WDP			
	Tertile	1st	2nd	3rd (+W)	1st	2nd	3rd (+T)	1st (+T)	2nd	3rd (+W)	Overall
	***N***	**Mean (SD)**	**Mean (SD)**	**Mean (SD)**	**Mean (SD)**	**Mean (SD)**	**Mean (SD)**	**Mean (SD)**	**Mean (SD)**	**Mean (SD)**	**Mean (SD)**
**Anthropometry**											
zBMI	272	**0.51 (1.26)**	0.47 (1.27)	**0.83 (1.19)**	0.70 (1.30)	0.68 (1.28)	0.35 (1.10)	0.40 (1.19)	0.64 (1.12)	0.74 (1.37)	0.60 (1.25)
***Bioelectrical impedance***											
%BF	260	21.5 (7.6)	21.6 (7.3)	23.3 (7.2)	**23.1 (7.8)**	22.4 (7.2)	**20.4 (7.0)**	**20.5 (7.4)**	22.4 (6.8)	**23.3 (7.7)**	22.1 (7.4)
**Lipid panel**											
TC (mg/dL)	272	169.1 (29.2)	169.7 (24.8)	169.2 (28.2)	171.5 (27.0)	168.3 (27.0)	168.1 (27.7)	170.5 (30.9)	169.2 (24.8)	168.9 (26.7)	169.4 (27.1)
LDL-c (mg/dL)	272	91.6 (25.0)	97.9 (24.8)	97.7 (24.9)	97.3 (26.0)	94.6 (24.8)	96.2 (24.1)	95.0 (26.4)	97.9 (23.1)	95.2 (25.8)	96.0 (24.9)
HDL-c (mg/dL)	272	61 (11)	59 (10)	58 (13)	59 (11)	60 (11)	58 (12)	60 (12)	59 (10)	59 (12)	59 (11)
TG (mg/dL)	272	63 (26)	62 (24)	63 (29)	65 (30)	61 (27)	62 (22)	63 (23)	62 (28)	63 (28)	63 (26)
ApoA1 (g/L)	272	**1.40 (0.24)**	1.34 (0.19)	**1.31 (0.21)**	1.35 (0.22)	1.34 (0.20)	1.36 (0.22)	**1.40 (0.23)**	1.35 (0.21)	**1.32 (0.20)**	1.35 (0.21)
ApoB (g/L)	272	0.72 (0.16)	0.74 (0.15)	0.74 (0.15)	0.74 (0.16)	0.72 (0.16)	0.73 (0.14)	0.74 (0.16)	0.74 (0.14)	0.72 (0.16)	0.73 (0.15)
TC/HDL	272	2.84 (0.59)	2.98 (0.69)	3.01 (0.6)	2.98 (0.66)	2.87 (0.65)	3.01 (0.63)	2.94 (0.64)	2.95 (0.66)	2.96 (0.65)	2.95 (0.65)
LDL/HDL	272	**1.56 (0.54)**	1.74 (0.64)	**1.77 (0.63)**	1.72 (0.63)	1.64 (0.61)	1.75 (0.61)	1.67 (0.61)	1.74 (0.61)	1.70 (0.63)	1.70 (0.61)
Apo B/ApoA1	272	0.55 (0.23)	0.56 (0.15)	0.57 (0.16)	0.57 (0.21)	0.56 (0.16)	0.55 (0.16)	0.55 (0.17)	0.58 (0.20)	0.56 (0.16)	0.56 (0.18)
LDL/ApoB	272	**126.6 (9.7)**	132.2 (14.4)	**132.0 (12.3)**	130.7 (11.8)	130.9 (13.7)	130.2 (12.5)	**127.3 (11.6)**	132.9 (13.6)	**131.9 (12.1)**	130.9 (12.7)
oxLDL-c (mU/L)	140	**5.79 (1.49)**	7.11 (1.34)	**6.97 (1.72)**	7.17 (1.59)	6.53 (1.82)	6.53 (1.41)	6.40 (1.69)	6.55 (1.12)	7.09 (1.83)	6.73 (1.61)
**Glycemic panel**											
Glucose (mg/dL)	272	82.9 (7.9)	82.1 (10.0)	80.2 (8.5)	80.4 (8.9)	81.8 (8.4)	83.0 (9.7)	83.1 (9.1)	81.6 (8.2)	79.2 (8.7)	81.7 (9.0)
Insulin (µU/mL)	140	3.44 (2.29)	3.73 (2.54)	3.98 (2.96)	3.50 (2.56)	4.11 (3.09)	3.50 (2.05)	3.66 (2.39)	3.68 (2.41)	3.88 (3.06)	3.75 (2.63)
HOMA-IR	140	0.66 (0.47)	0.73 (0.57)	0.70 (0.48)	0.65 (0.51)	0.76 (0.53)	0.67 (0.48)	0.73 (0.56)	0.70 (0.48)	0.68 (0.49)	0.70 (0.50)

Bold values highlight the statistically significant differences between first and third tertiles (*p* < 0.05). Results include sample size (N), mean and standard deviation (SD). +T closer to TDP, +W closer to WDP, WDP Western diet pattern, TDP Traditional diet pattern, TDP_WDP Western-Traditional combined score, ApoA1 apolipoprotein A1, ApoB apolipoprotein B, HDL-c high-density lipoprotein cholesterol, LDL-c low-density lipoprotein cholesterol, oxLDL-c LDL-c oxidised fraction, TC total cholesterol, TG triglycerides, HOMA-IR Homeostatic Model Assessment for Insulin Resistance, BF body fat, zBMI BMI *z*-score.

**Table 5:** Independent associations (*p*-value) between school- and child-level variables with zBMI, %BF, and metabolic risk factors through multilevel regression.

	Coefficient (*p*-Value)	zBMI	%BF	TG	TC	LDL/HDL	LDL/ApoB	HOMA-IR
**School-level**	VE	0%	0%	37%	1%	1%	2%	9%
Socio-economic								
	APPPC	0.000 (0.954)	0.010 (0.271)	−0.177 (0.604)	0.000 (0.995)	−0.001 (0.627)	0.009 (0.882)	−0.002 (0.533)
**Children-level**	VE	100%	100%	63%	100%	99%	98%	81%
Sex								
	Male	0.015 (0.890)	**−10.389 (0.046)**	−3.553 (0.177)	−1.037 (0.717)	**−0.155 (0.008)**	−0.597 (0.728)	−0.151 (0.264)
Diet scores								
	WDP	0.115 (0.127)	0.777 (0.092)	1.325 (0.448)	0.863 (0.648)	**0.093 (0.030) ^&^**	**2.378 (0.008) ^£^**	−0.014 (0.771)
	TDP	−0.120 (0.118)	−0.824 (0.084)	−1.576 (0.385)	0.123 (0.950)	0.016 (0.713)	0.421 (0.663)	−0.003 (0.952)
	TDP_WDP	**0.123 (0.025)**	**0.846 (0.013)**	1.425 (0.252)	0.390 (0.775)	0.066 (0.148)	0.986 (0.122)	−0.005 (0.880)
Extra-curricular PA								
	General	−0.046 (0.432)	−0.551 (0.120)	−1.622 (0.118)	−0.308 (0.831)	−0.015 (0.649)	0.315 (0.641)	−0.027 (0.445)
	Rest and leisure	0.052 (0.404)	0.167 (0.659)	−1.086 (0.426)	−1.856 (0.109)	0.029 (0.392)	−0.368 (0.601)	−0.029 (0.430)
	Prolonged	−0.017 (0.741)	−0.319 (0.299)	−0.754 (0.520)	0.357 (0.780)	−0.005 (0.864)	0.733 (0.210)	−0.046 (0.132)
	Intensive	0.003 (0.964)	−0.265 (0.553)	−4.778 (0.003) *	1.141 (0.527)	−0.032 (0.432)	0.227 (0.801)	−0.034 (0.457)
	Competition	0.017 (0.776)	−0.518 (0.140)	**−3.059 (0.003) ^#^**	−0.034 (0.981)	−0.041 (0.195)	−0.008 (0.991)	0.008 (0.830)
	Sports frequency	0.007 (0.435)	0.033 (0.548)	−0.082 (0.679)	−0.237 (0.280)	0.000 (0.999)	−0.064 (0.504)	−0.007 (0.207)
	Sports diversity	0.008 (0.821)	0.032 (0.873)	−0.366 (0.604)	−0.403 (0.607)	0.011 (0.545)	−0.213 (0.542)	−0.020 (0.304)

Coefficient’s significance: bold (*p* < 0.05); underline (*p* < 0.1). * **−3.629 (0.006)**, ^#^ **−2.612 (0.001)**, ^&^ 0.075 (0.077), and ^£^ **2.143 (0.025)** after adjusting for %BF and zBMI. WDP Western diet pattern, TDP Traditional diet pattern, TDP_WDP Western-Traditional combined score, PA Physical activity, VE % of total variance explained, APPPC Average purchasing power per capita. TC total cholesterol, TG triglycerides, ApoB apolipoprotein B, HDL-c high-density lipoprotein cholesterol, LDL-c low-density lipoprotein cholesterol, HOMA-IR Homeostatic Model Assessment for Insulin Resistance, BF body fat, zBMI BMI *z*-score.

## Data Availability

The datasets used and/or analysed during this study are available from the corresponding author upon reasonable request.

## Supplementary Materials

The following are available online at https://www.mdpi.com/article/10.3390/nu15020329/s1, Table S1: Questionnaire food items (by food groups).

## References

[1] Butte N.F. (2009). Impact of Infant Feeding Practices on Childhood Obesity. J. Nutr..

[2] Biro F.M., Wien M. (2010). Childhood Obesity and Adult Morbidities. Am. J. Clin. Nutr..

[3] Araújo J., Ramos E. (2017). Paediatric Obesity and Cardiovascular Risk Factors—A Life Course Approach. Porto Biomed. J..

[4] Chandrasekhar T., Suchitra M.M., Pallavi M., Srinivasa Rao P.V.L.N., Sachan A. (2017). Risk Factors for Cardiovascular Disease in Obese Children. Indian Pediatr..

[5] Elmaoğulları S., Tepe D., Uçaktürk S.A., Karaca Kara F., Demirel F. (2015). Prevalence of Dyslipidemia and Associated Factors in Obese Children and Adolescents. J. Clin. Res. Pediatr. Endocrinol..

[6] Reuter C.P., da Silva P.T., Renner J.D.P., de Mello E.D., Valim A.R.d.M., Pasa L., da Silva R., Burgos M.S. (2016). Dyslipidemia Is Associated with Unfit and Overweight-Obese Children and Adolescents. Arq. Bras. Cardiol..

[7] Bass R., Eneli I. (2015). Severe Childhood Obesity: An under-Recognised and Growing Health Problem. Postgrad. Med. J..

[8] Gupta N., Goel K., Shah P., Misra A. (2012). Childhood Obesity in Developing Countries: Epidemiology, Determinants, and Prevention. Endocr. Rev..

[9] Bentham J., Di Cesare M., Bilano V., Bixby H., Zhou B., Stevens G.A., Riley L.M., Taddei C., Hajifathalian K., Lu Y. (2017). Worldwide Trends in Body-Mass Index, Underweight, Overweight, and Obesity from 1975 to 2016: A Pooled Analysis of 2416 Population-Based Measurement Studies in 128.9 Million Children, Adolescents, and Adults. Lancet.

[10] Swallen K.C., Reither E.N., Haas S.A., Meier A.M. (2005). Overweight, Obesity, and Health-Related Quality of Life Among Adolescents: The National Longitudinal Study of Adolescent Health. Pediatrics.

[11] Feldman W., Feldman E., Goodman J.T. (1988). Culture versus Biology: Children’s Attitudes toward Thinness and Fatness. Pediatrics.

[12] Mustillo S., Worthman C., Erkanli A., Keeler G., Angold A., Costello E.J. (2003). Obesity and Psychiatric Disorder: Developmental Trajectories. Pediatrics.

[13] Strauss R.S. (2000). Childhood Obesity and Self-Esteem. Pediatrics.

[14] Cathaoir K.Ó., Abdeen Z.A., Hamid Z.A., Abu-Rmeileh N.M., Acosta-Cazares B., Acuin C., Adams R.J., Aekplakorn W., Afsana K., NCD Risk Factor Collaboration (NCD-RisC) (2016). Childhood Obesity and the Right to Health. Health Hum. Rights.

[15] Padez C., Mourão I., Moreira P., Rosado V. (2005). Prevalence and Risk Factors for Overweight and Obesity in Portuguese Children. Acta Paediatr..

[16] Aranceta-Bartrina J., Pérez-Rodrigo C. (2016). Determinants of Childhood Obesity: ANIBES Study. Nutr. Hosp..

[17] Buoncristiano M., Spinelli A., Williams J., Nardone P., Rito A.I., García-Solano M., Grøholt E.K., Gutiérrez-González E., Klepp K.I., Starc G. (2021). Childhood Overweight and Obesity in Europe: Changes from 2007 to 2017. Obes. Rev..

[18] Fismen A.-S., Buoncristiano M., Williams J., Helleve A., Abdrakhmanova S., Bakacs M., Bergh I.H., Boymatova K., Duleva V., Fijałkowska A. (2021). Socioeconomic differences in food habits among 6- to 9-year-old children from 23 countries—WHO European Childhood Obesity Surveillance Initiative (COSI 2015/2017). Obes. Rev..

[19] Musić Milanović S., Buoncristiano M., Križan H., Rathmes G., Williams J., Hyska J., Duleva V., Zamrazilová H., Hejgaard T., Jørgensen M.B. (2021). Socioeconomic Disparities in Physical Activity, Sedentary Behavior and Sleep Patterns among 6- to 9-Year-Old Children from 24 Countries in the WHO European Region. Obes. Rev..

[20] Isong I.A., Rao S.R., Bind M.-A., Avendaño M., Kawachi I., Richmond T.K. (2018). Racial and Ethnic Disparities in Early Childhood Obesity. Pediatrics.

[21] Ramiro-González M.D., Sanz-Barbero B., Royo-Bordonada M.Á. (2017). Childhood Excess Weight in Spain From 2006 to 2012. Determinants and Parental Misperception. Rev. Esp. Cardiol. Engl. Ed..

[22] Pietrobelli A., Pecoraro L., Ferruzzi A., Heo M., Faith M., Zoller T., Antoniazzi F., Piacentini G., Fearnbach S.N., Heymsfield S.B. (2020). Effects of COVID-19 Lockdown on Lifestyle Behaviors in Children with Obesity Living in Verona, Italy: A Longitudinal Study. Obesity.

[23] Neshteruk C.D., Zizzi A., Suarez L., Erickson E., Kraus W.E., Li J.S., Skinner A.C., Story M., Zucker N., Armstrong S.C. (2021). Weight-Related Behaviors of Children with Obesity during the COVID-19 Pandemic. Child. Obes..

[24] Stavridou A., Kapsali E., Panagouli E., Thirios A., Polychronis K., Bacopoulou F., Psaltopoulou T., Tsolia M., Sergentanis T.N., Tsitsika A. (2021). Obesity in Children and Adolescents during COVID-19 Pandemic. Children.

[25] Pires A., Martins P., Pereira A.M., Silva P.V., Marinho J., Marques M., Castela E., Sena C., Seiça R. (2015). Insulin Resistance, Dyslipidemia and Cardiovascular Changes in a Group of Obese Children. Arq. Bras. Cardiol..

[26] Chiarelli F., Marcovecchio M.L. (2008). Insulin resistance and obesity in childhood. Eur. J. Endocrinol..

[27] Turer C.B., Brady T.M., de Ferranti S.D. (2018). Obesity, Hypertension, and Dyslipidemia in Childhood Are Key Modifiable Antecedents of Adult Cardiovascular Disease: A Call to Action. Circulation.

[28] Leech R.M., McNaughton S.A., Timperio A. (2014). The Clustering of Diet, Physical Activity and Sedentary Behavior in Children and Adolescents: A Review. Int. J. Behav. Nutr. Phys. Act..

[29] Duffey K.J., Huybrechts I., Mouratidou T., Libuda L., Kersting M., De Vriendt T., Gottrand F., Widhalm K., Dallongeville J., Hallström L. (2012). Beverage Consumption among European Adolescents in the HELENA Study. Eur. J. Clin. Nutr..

[30] Vilela S., Correia D., Severo M., Oliveira A., Torres D., Lopes C., the IAN-AF Consortium (2019). Eating Frequency and Weight Status in Portuguese Children Aged 3–9 Years: Results from the Cross-Sectional National Food, Nutrition and Physical Activity Survey 2015–2016. Public Health Nutr..

[31] Vereecken C., Pedersen T.P., Ojala K., Krølner R., Dzielska A., Ahluwalia N., Giacchi M., Kelly C. (2015). Fruit and Vegetable Consumption Trends among Adolescents from 2002 to 2010 in 33 Countries. Eur. J. Public Health.

[32] López-Sobaler A.M., Aparicio A., Salas-González M.D., Loria Kohen V., Bermejo López L.M. (2021). Childhood Obesity in Spain and Associated Factors. Nutr. Hosp..

[33] Soon L., Gilliland J., Minaker L.M. (2022). Junk Food Accessibility After 10 Years of a Restrictive Food Environment Zoning Policy Around Schools. J. Am. Plan. Assoc..

[34] Flynn M.A.T., McNeil D.A., Maloff B., Mutasingwa D., Wu M., Ford C., Tough S.C. (2006). Reducing Obesity and Related Chronic Disease Risk in Children and Youth: A Synthesis of Evidence with ‘Best Practice’ Recommendations. Obes. Rev..

[35] Owen N., Healy G.N., Matthews C.E., Dunstan D.W. (2010). Too Much Sitting: The Population Health Science of Sedentary Behavior. Exerc. Sport Sci. Rev..

[36] LeBlanc A.G., Katzmarzyk P.T., Barreira T.V., Broyles S.T., Chaput J.-P., Church T.S., Fogelholm M., Harrington D.M., Hu G., Kuriyan R. (2015). Correlates of Total Sedentary Time and Screen Time in 9–11 Year-Old Children around the World: The International Study of Childhood Obesity, Lifestyle and the Environment. PLoS ONE.

[37] Owen N., Salmon J., Koohsari M.J., Turrell G., Giles-Corti B. (2014). Sedentary Behaviour and Health: Mapping Environmental and Social Contexts to Underpin Chronic Disease Prevention. Br. J. Sports Med..

[38] Carvalhal M.M., Padez M.C., Moreira P.A., Rosado V.M. (2007). Overweight and Obesity Related to Activities in Portuguese Children, 7–9 Years. Eur. J. Public Health.

[39] Cooper A.R., Goodman A., Page A.S., Sherar L.B., Esliger D.W., van Sluijs E.M.F., Andersen L.B., Anderssen S., Cardon G., Davey R. (2015). Objectively Measured Physical Activity and Sedentary Time in Youth: The International Children’s Accelerometry Database (ICAD). Int. J. Behav. Nutr. Phys. Act..

[40] Grieser M., Vu M.B., Bedimo-Rung A.L., Neumark-Sztainer D., Moody J., Young D.R., Moe S.G. (2006). Physical Activity Attitudes, Preferences, and Practices in African American, Hispanic, and Caucasian Girls. Health Educ. Behav..

[41] Vandenbroucke J.P., Von Elm E., Altman D.G., Gøtzsche P.C., Mulrow C.D., Pocock S.J., Poole C., Schlesselman J.J., Egger M. (2007). Strengthening the Reporting of Observational Studies in Epidemiology (STROBE): Explanation and Elaboration. Ann. Intern. Med..

[42] Lachat C., Hawwash D., Ocké M.C., Berg C., Forsum E., Hörnell A., Larsson C., Sonestedt E., Wirfält E., Åkesson A. (2016). Strengthening the Reporting of Observational Studies in Epidemiology—Nutritional Epidemiology (STROBE-Nut): An Extension of the STROBE Statement. PLoS Med..

[43] Almeida S.M., Furtado J.M., Mascarenhas P., Ferraz M.E., Silva L.R., Ferreira J.C., Monteiro M., Vilanova M., Ferraz F.P. (2016). Anthropometric Predictors of Body Fat in a Large Population of 9-Year-Old School-Aged Children. Obes. Sci. Pract..

[44] Cole T.J., Lobstein T. (2012). Extended International (IOTF) Body Mass Index Cut-Offs for Thinness, Overweight and Obesity. Pediatr. Obes..

[45] Furtado J.M., Almeida S.M., Mascarenhas P., Ferraz M.E., Ferreira J.C., Vilanova M., Monteiro M.P., Ferraz F.P. (2018). Anthropometric Features as Predictors of Atherogenic Dyslipidemia and Cardiovascular Risk in a Large Population of School-Aged Children. PLoS ONE.

[46] De Jesus J.M. (2011). Expert Panel on Integrated Guidelines for Cardiovascular Health and Risk Reduction in Children and Adolescents: Summary Report. Pediatrics.

[47] Gillum R.F. (2001). Indices of Adipose Tissue Distribution, Apolipoproteins B and AI, Lipoprotein (a), and Triglyceride Concentration in Children Aged 4-11 Years: The Third National Health and Nutrition Examination Survey. J. Clin. Epidemiol..

[48] Bachorik P.S., Lovejoy K.L., Carroll M.D., Johnson C.L. (1997). Apolipoprotein B and AI Distributions in the United States, 1988–1991: Results of the National Health and Nutrition Examination Survey III (NHANES III). Clin. Chem..

[49] Kurtoğlu S., Hatipoğlu N., Mazicioglu M.M., Kendirici M., Keskin M., Kondolot M. (2010). Insulin Resistance in Obese Children and Adolescents: HOMA-IR Cut-Off Levels in the Prepubertal and Pubertal Periods. J. Clin. Res. Pediatr. Endocrinol..

[50] Wijnhoven T.M.A., van Raaij J.M.A., Yngve A., Sjöberg A., Kunešová M., Duleva V., Petrauskiene A., Rito A.I., Breda J. (2015). WHO European Childhood Obesity Surveillance Initiative: Health-Risk Behaviours on Nutrition and Physical Activity in 6–9-Year-Old Schoolchildren. Public Health Nutr..

[51] Mota J., Santos P., Guerra S., Ribeiro J.C., Duarte J.A., Sallis J.F. (2002). Validation of a Physical Activity Self-Report Questionnaire in a Portuguese Pediatric Population. Pediatr. Exerc. Sci..

[52] Fundação Francisco Manuel Dos Santos (2013). Retrato de Portugal: Indicadores. https://www.pordata.pt/Municipios.

[53] Bates D., Mächler M., Bolker B., Walker S. (2015). Fitting Linear Mixed-Effects Models Using lme4. J. Stat. Softw..

[54] Rito A.I., Carvalho M.A., Ramos C., Breda J. (2013). Program Obesity Zero (POZ)—A Community-Based Intervention to Address Overweight Primary-School Children from Five Portuguese Municipalities. Public Health Nutr..

[55] Spinelli A., Buoncristiano M., Kovacs V.A., Yngve A., Spiroski I., Obreja G., Starc G., Pérez N., Rito A.I., Kunešová M. (2019). Prevalence of Severe Obesity among Primary School Children in 21 European Countries. Obes. Facts.

[56] Goiana-da-Silva F., Cruz-e-Silva D., Gregório M.J., Miraldo M., Darzi A., Araújo F. (2018). The Future of the Sweetened Beverages Tax in Portugal. Lancet Public Health.

[57] Shinn C., Salgado R., Rodrigues D. (2020). National Programme for Promotion of Physical Activity: The Situation in Portugal. Cien. Saude Colet..

[58] Pereira M., Nogueira H., Gama A., Machado-Rodrigues A., Rosado-Marques V., Silva M.-R.G., Padez C. (2021). The Economic Crisis Impact on the Body Mass Index of Children Living in Distinct Urban Environments. Public Health.

[59] Rito A., Graça P. Childhood Obesity Surveillance Initiative: COSI Portugal 2013 Report. http://hdl.handle.net/10400.18/3108.

[60] Moniz M., Marques T., Cabral M., Nizarali Z., Coelho R., Monteiro A., Bragança G., Carreiro H. (2011). Risco Cardiovascular Na Obesidade Infantil. Acta Med. Port..

[61] Vera S., Figueroa J.T., Aranzález L.H., Mockus I. (2016). Nutritional Status and Cardiovascular Risks in Children of Two Schools in Bogotá, Colombia. Arch. Latinoam. Nutr..

[62] I’Allemand D., Wiegand S., Reinehr T., Müller J., Wabitsch M., Widhalm K., Holl R., APV-Study Group (2008). Cardiovascular Risk in 26,008 European Overweight Children as Established by a Multicenter Database. Obesity.

[63] Nascimento H., Costa E., Rocha-Pereira P., Rego C., Mansilha H.F., Quintanilha A., Santos-Silva A., Belo L. (2012). Cardiovascular Risk Factors in Portuguese Obese Children and Adolescents: Impact of Small Reductions in Body Mass Index Imposed by Lifestyle Modifications. Open Biochem. J..

[64] Castorani V., Polidori N., Giannini C., Blasetti A., Chiarelli F. (2020). Insulin Resistance and Type 2 Diabetes in Children. Ann. Pediatr. Endocrinol. Metab..

[65] Mayer-Davis E.J., Lawrence J.M., Dabelea D., Divers J., Isom S., Dolan L., Imperatore G., Linder B., Marcovina S., Pettitt D.J. (2017). Incidence Trends of Type 1 and Type 2 Diabetes among Youths, 2002–2012. N. Engl. J. Med..

[66] Candler T.P., Mahmoud O., Lynn R.M., Majbar A.A., Barrett T.G., Shield J.P.H. (2018). Continuing Rise of Type 2 Diabetes Incidence in Children and Young People in the UK. Diabet. Med..

[67] Conwell L.S., Trost S.G., Brown W.J., Batch J.A. (2004). Indexes of Insulin Resistance and Secretion in Obese Children and Adolescents: A Validation Study. Diabetes Care.

[68] Keskin M., Kurtoglu S., Kendirci M., Atabek M.E., Yazici C. (2005). Homeostasis Model Assessment Is More Reliable than the Fasting Glucose/Insulin Ratio and Quantitative Insulin Sensitivity Check Index for Assessing Insulin Resistance among Obese Children and Adolescents. Pediatrics.

[69] Jeffery A.N., Metcalf B.S., Hosking J., Streeter A.J., Voss L.D., Wilkin T.J. (2012). Age Before Stage: Insulin Resistance Rises Before the Onset of PubertyA 9-Year Longitudinal Study (EarlyBird 26). Diabetes Care.

[70] Shalitin S., Gat-Yablonski G. (2022). Associations of Obesity with Linear Growth and Puberty. Horm. Res. Paediatr..

[71] Kaplowitz P.B. (2008). Link Between Body Fat and the Timing of Puberty. Pediatrics.

[72] Peplies J., Börnhorst C., Günther K., Fraterman A., Russo P., Veidebaum T., Tornaritis M., De Henauw S., Marild S., Molnar D. (2016). Longitudinal Associations of Lifestyle Factors and Weight Status with Insulin Resistance (HOMA-IR) in Preadolescent Children: The Large Prospective Cohort Study IDEFICS. Int. J. Behav. Nutr. Phys. Act..

[73] Domínguez M., Vega-Hernández M.C., De la Calle Cabrera T., Arriba-Méndez S., Pellegrini-Belinchón F.J. (2022). Mediterranean Diet in the Castilian Plains: Dietary Patterns and Childhood Asthma in 6–7-Year-Old Children from the Province of Salamanca. Allergol. Immunopathol..

[74] Arcila-Agudelo A.M., Ferrer-Svoboda C., Torres-Fernàndez T., Farran-Codina A. (2019). Determinants of Adherence to Healthy Eating Patterns in a Population of Children and Adolescents: Evidence on the Mediterranean Diet in the City of Mataró (Catalonia, Spain). Nutrients.

[75] Davison B., Saeedi P., Black K., Harrex H., Haszard J., Meredith-Jones K., Quigg R., Skeaff S., Stoner L., Wong J.E. (2017). The Association between Parent Diet Quality and Child Dietary Patterns in Nine- to Eleven-Year-Old Children from Dunedin, New Zealand. Nutrients.

[76] Huybrechts I., Lioret S., Mouratidou T., Gunter M.J., Manios Y., Kersting M., Gottrand F., Kafatos A., De Henauw S., Cuenca-García M. (2017). Using Reduced Rank Regression Methods to Identify Dietary Patterns Associated with Obesity: A Cross-Country Study among European and Australian Adolescents. Br. J. Nutr..

[77] Kourlaba G., Panagiotakos D.B., Mihas K., Alevizos A., Marayiannis K., Mariolis A., Tountas Y. (2009). Dietary Patterns in Relation to Socio-Economic and Lifestyle Characteristics among Greek Adolescents: A Multivariate Analysis. Public Health Nutr..

[78] Afeiche M.C., Taillie L.S., Hopkins S., Eldridge A.L., Popkin B.M. (2017). Breakfast Dietary Patterns among Mexican Children Are Related to Total-Day Diet Quality. J. Nutr..

[79] Del Mar Bibiloni M., Martílnez E., Llull R., Pons A., Tur J.A. (2012). Western and Mediterranean Dietary Patterns among Balearic Islands’ Adolescents: Socio-Economic and Lifestyle Determinants. Public Health Nutr..

[80] Joung H., Hong S., Song Y., Ahn B.C., Park M.J. (2012). Dietary Patterns and Metabolic Syndrome Risk Factors among Adolescents. Korean J. Pediatr..

[81] Yoo Y.-J., Oh J.-H., Lee W.C., Woo K.M. (2016). Regenerative Characteristics of Apical Papilla–derived Cells from Immature Teeth with Pulpal and Periapical Pathosis. J. Endod..

[82] Funtikova A.N., Navarro E., Bawaked R.A., Fíto M., Schröder H. (2015). Impact of Diet on Cardiometabolic Health in Children and Adolescents. Nutr. J..

[83] Shively C.A., Appt S.E., Vitolins M.Z., Uberseder B., Michalson K.T., Silverstein-Metzler M.G., Register T.C. (2019). Mediterranean versus Western Diet Effects on Caloric Intake, Obesity, Metabolism, and Hepatosteatosis in Nonhuman Primates. Obesity.

[84] Nicklas T.A., Dwyer J., Feldman H.A., Luepker R.V., Kelder S.H., Nader P.R. (2002). Serum Cholesterol Levels in Children Are Associated with Dietary Fat and Fatty Acid Intake. J. Am. Diet. Assoc..

[85] Bibiloni M.M., Martínez E., Llull R., Maffiotte E., Riesco M., Llompart I., Pons A., Tur J.A. (2011). Metabolic Syndrome in Adolescents in the Balearic Islands, a Mediterranean Region. Nutr. Metab. Cardiovasc. Dis..

[86] George E.S., Gavrili S., Itsiopoulos C., Manios Y., Moschonis G. (2021). Poor Adherence to the Mediterranean Diet Is Associated with Increased Likelihood of Metabolic Syndrome Components in Children: The Healthy Growth Study. Public Health Nutr..

[87] Klish W.J., Baker S.S., Cochran W.J., Flores C.A., Georgieff M.K., Jacobson M.S., Lake A., Harris S.S., Hubbard V.S., Levin E. (1998). Cholesterol in Childhood. Pediatrics.

[88] Kelley G.A., Kelley K.S. (2007). Aerobic Exercise and Lipids and Lipoproteins in Children and Adolescents: A Meta-Analysis of Randomized Controlled Trials. Atherosclerosis.

[89] Cesa C.C., Molino G.O.G., Lima J., Pereira R.B., Eibel B., Barbiero S.M., Schaan B.D., Pellanda L.C. (2022). Physical Activity and Cardiovascular Risk Factors in Children: A Meta-Analysis Update. Int. J. Cardiovasc. Sci..

