# Supplementary material for: Pediatric Overweight, Fatness and Risk for Dyslipidemia Are Related to Diet: A Cross-Sectional Study in 9-year-old Children
Source: Nutrients. 2023 Jan 9;15(2):329. doi: 10.3390/nu15020329 (PMC9865454; doi:10.3390/nu15020329)
Supplement: Supplementary file 1 [file nutrients-15-00329-s001.zip › nutrients-2121200-supplementary.pdf]

Table S1 – Questionnaire food items (by food groups)

Food groups (17)	Food items (33)
Chips and salt sticks	salty snacks, crisps and chips
Compound fast food	pizza, outdoor hamburger
Processed meat	sausage, jambon
Sweets	candies, gums and chocolates, jam
Bread	bread, toasts
Oils and fats	butter or margarine
Red meat and poultry	meat, home-made hamburger
Fish	fish
Soup	vegetable soup
Fruits and fresh fruits juice	fresh fruit, fresh fruit juices
Water	water
Vegetables	vegetable salads
Fried potatoes	fried potatoes
Cake and cookies	biscuits, cookies and cakes
Sugar-sweetened beverages	sweet beverages, sweet teas
Cereals	cereals
Milk and dairy products	cheese, milk, milk with chocolate, milk with coffee, yogurt, yogurt with fruit or aroma, lacteal products

Methods

Anthropometric and bioelectrical impedance analysis

Clinical procedures were carried out at school under the guidance of two pediatric specialists, which had previously been assessed for the equivalence of their measuring performance. All anthropometric measurements were taken with participants dressed in lightweight clothing and without shoes. Height was measured to the nearest 0.1 cm at the end of a deep inspiration (stadiometer – Seca 217, Hamburg, Deutschland). Weight was measured to the nearest 0.1 kg (digital calibrated scale – Seca 899). The circumference measurements were taken to the nearest 0.1 cm, with the tape snug but not compressing the skin, and were made using flexible and inextensible tape (Seca 203). MUAC was measured on the upper left arm, flexed at 90°, at the midpoint between the acromion and the olecranon. CC was measured at the point of the widest diameter of the calf. Both measurements were taken while participants were sitting. WC and HC were measured in the standing position, with the first at the umbilicus level at the end of normal expiration and the second at the widest part of the hip at the level of the great trochanter. Waist–hip ratio (WHR) was defined as WC/HC. WHtR was defined as WC/height. Tetrapolar whole-body BIA (measured with Omron BF 511 – 500 mÅ, 50 kHz, Kyoto, Japan) was used to evaluate %BF, percentage of skeletal muscle (%SM), and resting metabolic rate (RMR), all of which have been validated previously in children against Magnetic Resonance Imaging findings. Children were measured 2 hours or more after breakfast according to the manufacturer's instructions.

Biochemical analysis

Participants were asked to fast for 12 hours, and whole blood was collected by venipuncture in the morning at school. Blood samples were refrigerated at 5°C until processed for serum separation. The serum aliquots were frozen (-80°C) and stored until further analysis. Serum samples were assessed for TC, HDL-c, LDL-c and TG, as well as ApoA1 and ApoB, using turbidimetric immuno-enzymatic assays. Serum samples were tested for glucose (Glucose oxidase method). All biochemical assessments were performed using *Horiba Medical* reagents in a *Horiba Pentra C400* auto-analyzer (France. Obtained intra and inter-assay coefficients of variation were <3%. Insulin (kit—10-1113-01, Mercodia) and oxLDL-c (kit– 10-1143-01, Mercodia) were evaluated by ELISA in a DS2 auto-analyzer from *Dynex Technologies*™ (Magellan Biosciences, USA).
